# Ergosterol distribution controls surface structure formation and fungal pathogenicity

**DOI:** 10.1128/mbio.01353-23

**Published:** 2023-07-06

**Authors:** Hau Lam Choy, Elizabeth A. Gaylord, Tamara L. Doering

**Affiliations:** 1 Department of Molecular Microbiology, Washington University School of Medicine, St. Louis, Missouri, USA; Duke University Hospital, Durham, North Carolina, USA

**Keywords:** *Cryptococcus neoformans*, Ysp2, sterol transport, ergosterol, virulence, mycology

## Abstract

Ergosterol, the major sterol in fungal membranes, is critical for defining membrane fluidity and regulating cellular processes. Although ergosterol synthesis has been well defined in model yeast, little is known about sterol organization in the context of fungal pathogenesis. We identified a retrograde sterol transporter, Ysp2, in the opportunistic fungal pathogen *Cryptococcus neoformans*. We found that the lack of Ysp2 under host-mimicking conditions leads to abnormal accumulation of ergosterol at the plasma membrane, invagination of the plasma membrane, and malformation of the cell wall, which can be functionally rescued by inhibiting ergosterol synthesis with the antifungal drug fluconazole. We also observed that cells lacking Ysp2 mislocalize the cell surface protein Pma1 and have abnormally thin and permeable capsules. As a result of perturbed ergosterol distribution and its consequences, *ysp2*∆ cells cannot survive in physiologically relevant environments such as host phagocytes and are dramatically attenuated in virulence. These findings expand our knowledge of cryptococcal biology and underscore the importance of sterol homeostasis in fungal pathogenesis.

**IMPORTANCE:**

*Cryptococcus neoformans* is an opportunistic fungal pathogen that kills over 100,000 people worldwide each year. Only three drugs are available to treat cryptococcosis, and these are variously limited by toxicity, availability, cost, and resistance. Ergosterol is the most abundant sterol in fungi and a key component in modulating membrane behavior. Two of the drugs used for cryptococcal infection, amphotericin B and fluconazole, target this lipid and its synthesis, highlighting its importance as a therapeutic target. We discovered a cryptococcal ergosterol transporter, Ysp2, and demonstrated its key roles in multiple aspects of cryptococcal biology and pathogenesis. These studies demonstrate the role of ergosterol homeostasis in *C. neoformans* virulence, deepen our understanding of a pathway with proven therapeutic importance, and open a new area of study.

## INTRODUCTION

*Cryptococcus neoformans* is a fungal pathogen that causes 112,000 HIV-associated deaths per year and accounts for 19% of AIDS-related mortality (1). During infection, spores or desiccated yeast cells are inhaled, resulting in pulmonary infection. In immunocompetent hosts, cryptococcal infections are generally asymptomatic and are either cleared or remain latent. However, in immunocompromised patients, the fungi disseminate from the lungs and enter the central nervous system, resulting in often-fatal meningoencephalitis (2 - 4).

Ergosterol is the most abundant sterol in fungal membranes (5). It is critical in defining membrane fluidity and permeability and regulating protein sorting and the activity of membrane-associated enzymes (5, 6). Beyond basic biology, ergosterol is also an important therapeutic target for *C. neoformans* infections. Treatment options for cryptococcosis are limited to three drugs: amphotericin B (AmB), fluconazole, and flucytosine (7, 8). Of these, AmB and fluconazole target ergosterol itself or its biosynthetic pathway (9 - 11). Ergosterol synthesis has been well defined in model yeast *Saccharomyces cerevisiae* (6, 12), but less is known about sterol organization, particularly in the context of fungal pathogenesis.

**Fig 1:**
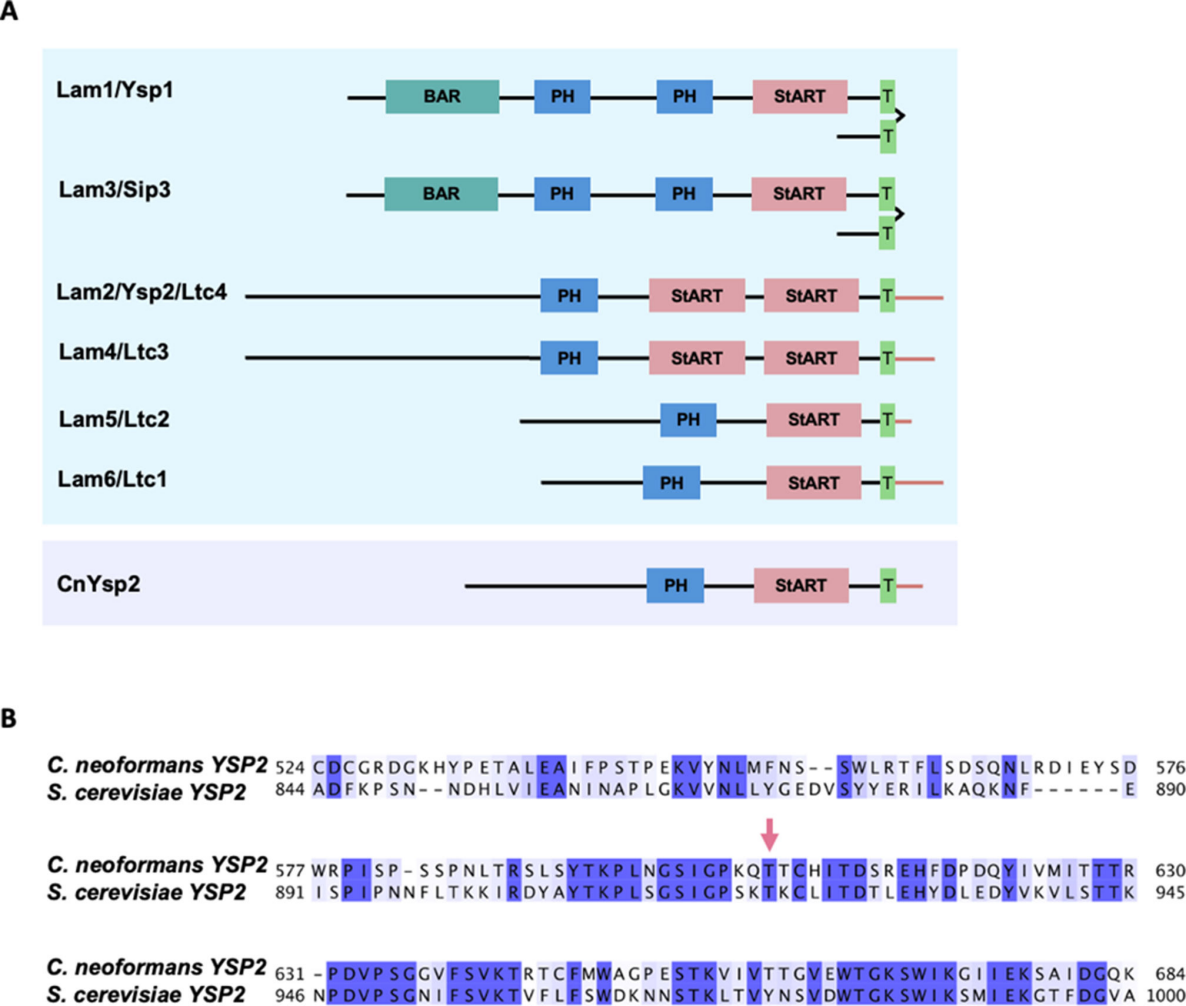
Alignment of cryptococcal Ysp2 with *S. cerevisiae* homologs. (**A**) LAM family proteins in *S. cerevisiae* (blue box) and *C. neoformans* (purple box). Domain abbreviations are Bar, Bin/amphiphysin/RVS; PH, pleckstrin-homology; StART, Steroidogenic Acute Regulatory Transfer-like; and T, transmembrane. (**B**) Alignment of *C. neoformans* and *S. cerevisiae YSP2* StART-like domains, with CLUSTALX coloring of conserved residues. Pink arrow, residue predicted to make van der Waals contact with ergosterol (22).

Although sterols are synthesized in the endoplasmic reticulum (ER), most are transported to other organelles, notably the plasma membrane (PM), which contains up to 90% of cellular sterols (13, 14). This movement is mainly mediated by sterol-specific lipid transport proteins, which are independent of the secretory pathway (15). *S. cerevisiae* expresses two families of these proteins: oxysterol-binding proteins (OSH) and lipid transfer proteins anchored at membrane contact sites (LAM). The seven cytosolic OSH proteins move sterols to and from the ER in exchange for other lipids (16 - 18). The recently identified LAM proteins are anchored by transmembrane domains and possess characteristic StART (Steroidogenic Acute Regulatory Transfer)-like domains that bind sterols (Fig. 1A) (19). In *S. cerevisiae,* this family is represented by six proteins, Lam1–Lam6 (19). Lam1–4 are localized at sites of ER-PM membrane contact, while Lam5 and Lam6 are localized at sites of contact between the ER and mitochondria or vacuoles (19, 20). Lam2, also called Ysp2, has been suggested to be a retrograde sterol transporter that moves sterols from the PM to the ER and is important for mitochondrial morphology (19, 21).

Mechanisms of sterol transport have been examined in model yeast but not in fungal pathogens, and the relationship between sterol organization and fungal pathogenesis remains unexplored. To tackle these questions, we investigated the role of the only apparent LAM family member in *C. neoformans*. This protein, named Ysp2 for its homology to the *S. cerevisiae* transporter, was previously identified in a caspofungin sensitivity screen and shown to influence membrane integrity (23). We found that lack of Ysp2 under conditions that mimic the mammalian host environment leads to excess accumulation of ergosterol at the PM, invagination of the PM, and striking malformation of the cell wall. These processes can be functionally rescued by inhibiting ergosterol synthesis with fluconazole. We also observed perturbations of sterol synthesis and storage in *ysp2*∆ mutant cells. We conclude that Ysp2 is a retrograde sterol transporter that is critical for survival in host environments and cryptococcal virulence.

## RESULTS

### Ysp2 is required for *in vivo* and *in vitro* virulence

We used the *S. cerevisiae* Ysp2 protein sequence to identify the homologous cryptococcal gene, *CNAG_00650*. BLASTp searches of the *S. cerevisiae* genome using this sequence yield the original *S. cerevisiae* gene; based on this reciprocity the *C. neoformans* protein has the same name. The Ysp2 proteins in *C. neoformans* and *S. cerevisiae* have 39% amino acid identity overall, with an E value of 6 × 10^−51^ (Fig. 1B shows homology in the StART-like domain). For functional studies, we generated a *ysp2*Δ deletion mutant in *C. neoformans* strain KN99α (referred to as wild type [WT] below) and also complemented it at the native locus (referred to as *YSP2*).

**Fig 2:**
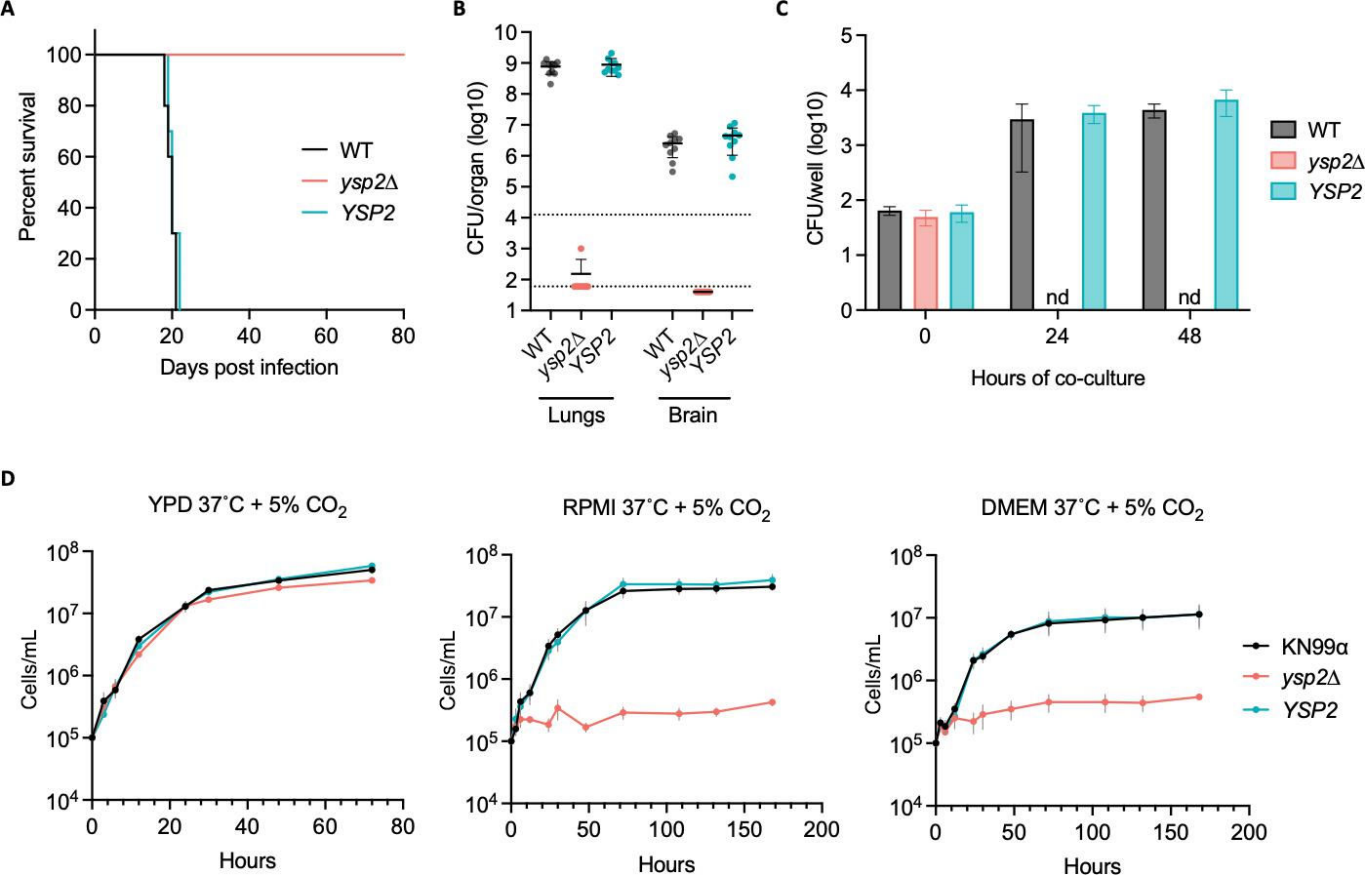
Ysp2 is required for virulence and survival in physiological environments. (**A**) Survival of C57BL/6 mice after intranasal infection with 1.25 × 10^4^ fungal cells, with sacrifice triggered by weight below 80% of initial weight. (**B**) Lung and brain fungal burdens of mice from Panel A at sacrifice. Top dotted line, initial inoculum; bottom dotted line, limit of detection. (**C**) *In vitro* survival of cryptococci. Bone marrow-derived macrophages were co-incubated with the indicated strains (1.5 h, MOI = 0.1) and washed to remove free fungi before lysis at the times shown and assessment of cryptococcal CFU. nd, not detected. Mean ± SD is plotted; results shown are representative of at least two biological replicate experiments. (**D**) Growth curves in the conditions shown (mean ± SEM of three independent experiments). YPD, yeast extract-peptone-dextrose medium; DMEM, Dulbecco's Modified Eagle Medium.

To investigate the role of Ysp2 in fungal pathogenesis, we assessed the virulence of *ysp2*∆ in a mouse model of cryptococcosis, where disease progression is monitored by weight loss. All mice infected with WT or complemented strains steadily lost weight (Fig. S1A) and succumbed to infection by day 22, but mice infected with *ysp2*∆ showed no signs of illness and were only sacrificed when the experiment was terminated at day 80 (Fig. 2A). Consistent with these findings, the lungs and brains of mice infected with WT or complemented strains showed high fungal burden at sacrifice, while those of mice infected with *ysp2*∆ yielded minimal fungi (Fig. 2B).

*C. neoformans* is a facultative intracellular pathogen, which may enter and survive within host phagocytes (24, 25). To determine the effect of Ysp2 on these processes, we examined fungal interactions with host macrophages. We found that *ysp2*∆ cells were phagocytosed at the same rate as the WT and complemented strains (Fig. 2C; Fig. S1B) but were much more susceptible to killing after internalization; they were completely cleared by 24 h of incubation, while the control populations significantly increased in that interval (Fig. 2C).

We wondered whether the severe attenuation of *ysp2*∆ cells in mice and macrophages reflected susceptibility to features of the host environment, independent of specific host responses. Upon testing this, we found that mutant growth in rich medium was not perturbed by exposure to host physiological temperature (37°C) or CO_2_ level (5%) (Fig. 2D; Fig. S2). However, the population no longer increased when the conditions were changed to incorporate mammalian tissue culture medium along with these environmental changes (Fig. 2D). Notably, the *ysp2*∆ cells remained viable under these conditions at all times shown, as demonstrated by their ability to form colonies upon transfer to rich medium (Fig. S1C).

### *C. neoformans* lacking Ysp2 exhibits defects in surface structures

**Fig 3:**
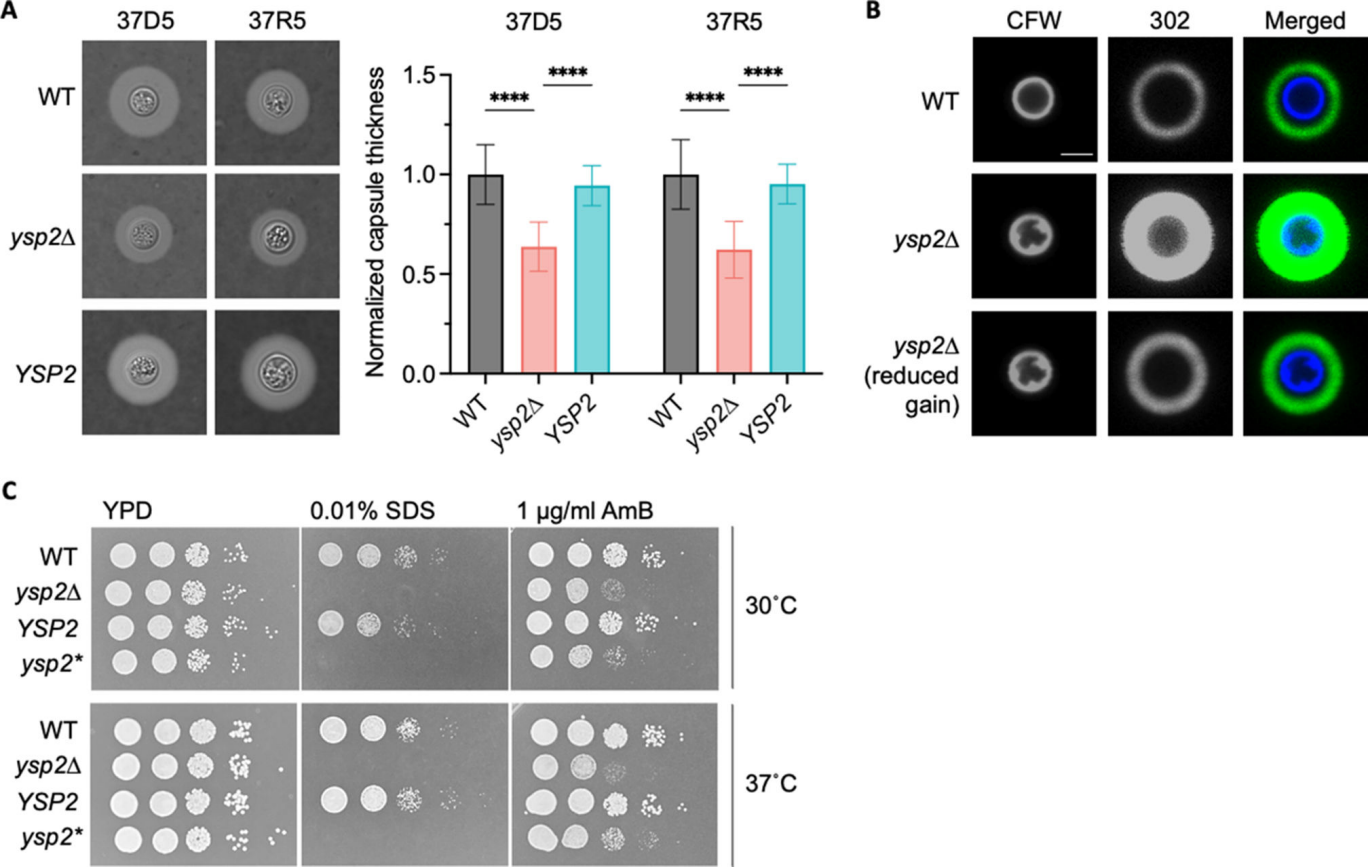
The *ysp2*∆ mutant exhibits cell surface defects. (**A**) Capsule thickness. The indicated strains were grown in host-like conditions [37°C, 5% CO_2_, with either DMEM (37D5) or RPMI (37R5)], stained with India ink (left), and capsule thickness was measured with ImageJ and normalized to cell radius and WT value (right). Mean ± SD of at least 50 cells per sample are shown. ****P < 0.0001 by one-way analysis of variance. (**B**) Representative confocal micrographs of the indicated strains after growth in 37R5 for 24  h and staining with CFW (cell wall) and MAb 302 conjugated to Alexa Fluor 488 (capsule). Images in the first and second rows were obtained at the same gain and intensity settings; in the third row, confocal gain of the α-capsule Ab channel was reduced (reduced gain). All images are to the same scale; bar, 5  µm. (**C**) Serial 10-fold dilutions of the indicated strains in the conditions shown. ysp2*, inactivated mutant (T606D) with abrogated sterol-binding activity.

To explore factors that might influence the pathogenicity of *C. neoformans* lacking Ysp2, we first examined the best-known cryptococcal virulence factor, its polysaccharide capsule. This structure presents a physical barrier to phagocytosis and modulates the host immune response (25 - 27). For cells grown in host-like conditions (which in this paper are modeled by mammalian tissue culture medium, 37°C, and 5% CO_2_), the capsule thickness of *ysp2*∆ was reduced by almost 40% compared to control strains (Fig. 3A). Interestingly, despite their thinner capsules, the mutant cells bound more anticapsule antibodies (Fig. 3B). These apparently contradictory results suggested a possible change in the mutant capsule architecture. We tested this idea by measuring the permeability of capsule to 2,000 kDa fluorescent dextran beads, using cell wall staining with calcofluor white (CFW) to mark its inner boundary. The beads penetrated twice as deeply into the capsule of *ysp2*∆ cells compared to WT (Fig. S3A), supporting our hypothesis. These studies also revealed some intriguing irregularities in the mutant cell wall compared to the smooth wall of WT cells (Fig. 3B, CFW staining); these are pursued below.

In *S. cerevisiae*, Ysp2 mediates retrograde sterol transport from the PM. If the cryptococcal homolog performs the same function, its absence may alter lipid distribution and thereby compromise membrane integrity. To test this hypothesis, we subjected mutant cells to membrane stress. For this, we plated serial dilutions of *ysp2*∆ cells in the presence of the detergent SDS or the antifungal compound AmB, which perturb membranes by solubilizing lipids and binding ergosterol, respectively. Compared to controls, *ysp2*∆ was far more sensitive to SDS (Fig. 3C). It was also more sensitive than control strains to AmB (Fig. 3C; Fig. S3B (28)), as was previously observed in *C. neoformans* (23) and in the corresponding *S. cerevisiae* mutant (19). These results are consistent with perturbed lipid organization in the mutant.

We suspected that the aberrant phenotypes of *ysp2*∆ were due to its inability to appropriately distribute sterols. In *S. cerevisiae*, residue T921 of Ysp2 is required for ergosterol binding and consequent retrograde transfer activity (22). We used Clustal Omega to identify T606 as the corresponding amino acid in *C. neoformans* and mutated the *YSP2* gene to replace this residue with aspartic acid (Fig. 1B). The resulting strain, ysp2*, phenocopied the deletion mutant *ysp2*∆ (Fig. 3C), supporting our model that defective ergosterol binding causes the observed phenotypes and attenuated virulence.

Sterol composition is also critical for the biogenesis and maintenance of mitochondrial membranes (29, 30), and Ysp2 has been implicated in mitochondrial morphology in model yeast (21). When we stained cells with MitoTracker CMXRos to assess *C. neoformans* mitochondrial morphology, we saw bright staining that was absent in WT (Fig. S4A). Because the accumulation of this compound depends on mitochondrial membrane potential, we wondered whether this characteristic was altered in *ysp2*∆. When we stained the mutant with tetramethylrhodamine ethyl ester (TMRE), a cationic dye that accumulates in mitochondrial inner membranes based on membrane potential, we observed a broad peak, with roughly 50% of cells exceeding control staining (Fig. S4B). However, *ysp2*∆ cells exhibited no growth defects on media containing alternative carbon sources or electron transport chain-inhibiting compounds (Fig. S4C (31)). We conclude that despite its effects on mitochondrial membranes, Ysp2 has minimal impact on mitochondrial function.

**Fig 4:**
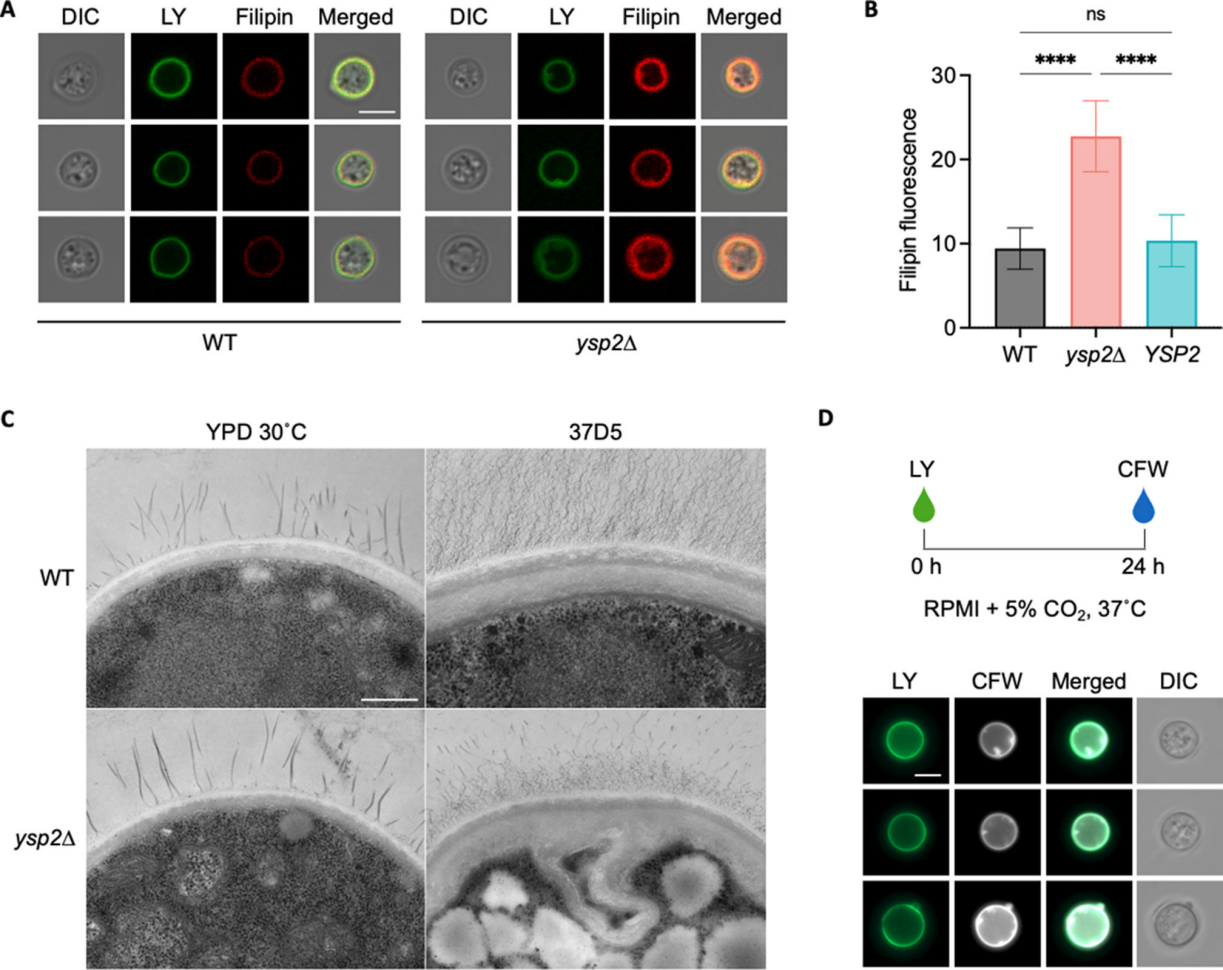
The *ysp2*∆ mutant exhibits malformations of the cell wall and plasma membrane. (**A**) Representative cells that were grown in 37R5 for 24 h and stained with Lucifer Yellow (LY) for cell wall and filipin for non-esterified sterols. All images are to the same scale; bar, 5 µm. DIC, differential interference contrast. (**B**) Filipin fluorescence (mean ± SD of mean gray value for at least 50 cells per sample). ****P < 0.0001 by one-way analysis of variance. (**C**) Transmission electron micrographs of cells grown in 37D5. All images are to the same scale; bar, 500 nm. (**D**) Representative *ysp2∆* cells that were stained with LY, grown in 37R5 for 24 h, and stained with CFW. All images are to the same scale; bar, 5 µm.

Based on Ysp2’s putative role in retrograde transport of sterols from the PM, we next focused our attention on this structure. To examine surface morphology, we stained cells with filipin, a fluorescent dye which binds sterols, and the cell wall dye Lucifer Yellow. Compared to the smooth ring staining patterns of control strains, *ysp2*∆ cells grown in host-like conditions showed irregular invaginations in both filipin and Lucifer Yellow signal (Fig. 4A), similar to what we had noted earlier with CFW (Fig. 3B). The mutant cells additionally showed brighter filipin fluorescence (Fig. 4A and B); this suggested higher sterol levels, which would also be consistent with impaired sterol removal from the plasma membrane. For a more detailed view of this striking phenotype, we examined the cells using transmission electron microscopy. Corroborating our light microscopy results, WT cells displayed even curvature of the cell wall and underlying PM. In contrast, the mutant showed distorted areas of both structures, which were only present when the cells were grown in host-like conditions (Fig. 4C). Furthermore, in some regions, layers of wall material appeared to surround both membranous material and cytoplasmic content (Fig. 4C).

Intrigued by the unusual surface deformations of cells lacking Ysp2, we examined the kinetics of their formation. To do this, we grew cells in rich medium, stained their cell walls with Lucifer Yellow, cultured them in host-like conditions for 24 h, and then stained them with CFW. In these studies, the Lucifer Yellow introduced before the culture period occurred as a smooth ring (Fig. 4D); the surface invaginations were evident only in the CFW staining following 24 h of growth. This suggests that the deformed regions are composed of newly synthesized cell wall material produced during the period of growth in host-like conditions, rather than being composed of older cell wall material that was somehow rearranged.

### Protein localization

**Fig 5:**
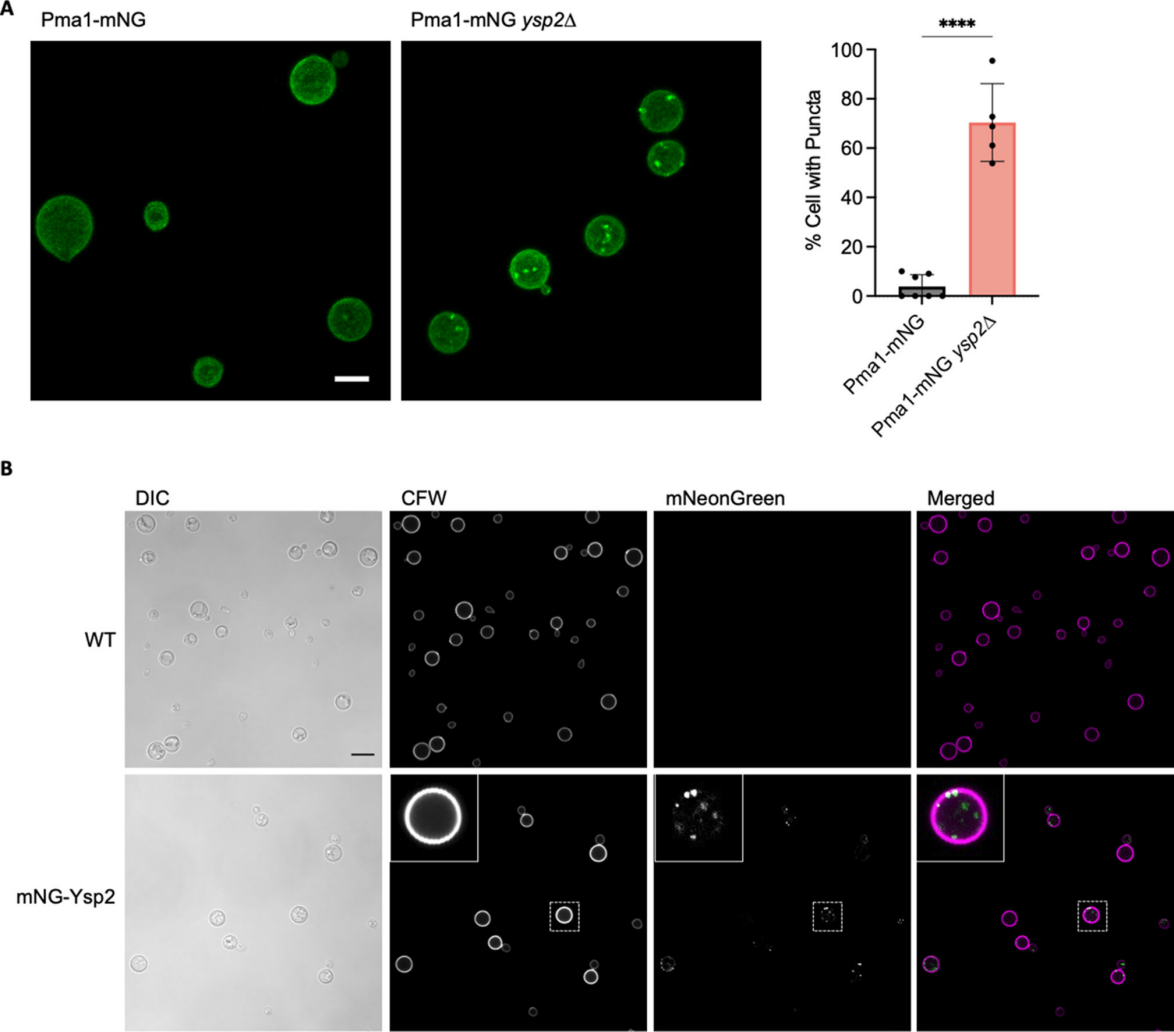
Protein localization imaged by confocal microscopy. (**A**) Pma1-mNeonGreen (Pma1-mNG) expressed in WT and *ysp2∆* cells. Images, maximum intensity projections that sum Z-stacks. Both are to the same scale; bar, 5 µm. Plot, percent of cells with fluorescent puncta (mean ± SD based on at least 50 cells across five to seven image fields). ****P < 0.0001 by Student’s t test. (**B**) Fluorescence and DIC images of WT cells alone (top) or the same cells expressing mNeonGreen-Ysp2 (mNG-Ysp2, bottom). Inset, an example cell (boxed) enlarged threefold. Bar, 5 µm.

Lipid rafts, or detergent-resistant microdomains, are ordered domains of the PM that are enriched in sphingolipids, sterols, and GPI-anchored polypeptides (32). In *C. neoformans,* one raft protein is Pma1, a PM ATPase that is required for survival within host cells (33). To determine whether sterol accumulation in the PM would affect localization of resident proteins, we generated strains expressing Pma1-mNeonGreen from the endogenous locus in both WT and *ysp2*∆ backgrounds. Although Pma1 normally localizes primarily to the PM in a uniform pattern, in *ysp2*∆ cells it also appeared as bright puncta (Fig. 5A). This pattern occurred in roughly 70% of *ysp2*∆ cells, compared to 4% of WT (Fig. 5A), although gene expression was similar in both backgrounds (Fig. S5A).

We also assessed the subcellular distribution of Ysp2 itself, by engineering cells to express mNeonGreen-Ysp2 from the native locus (Fig. 5B). In addition to puncta of fluorescent protein at the cell periphery (compare to CFW staining), we observed roughly 35% (± SD of 10%) of the Ysp2 signal within the cell, quite different from its homolog in model yeast (see Discussion).

### Ysp2 modulates sterol distribution and abundance

**Fig 6:**
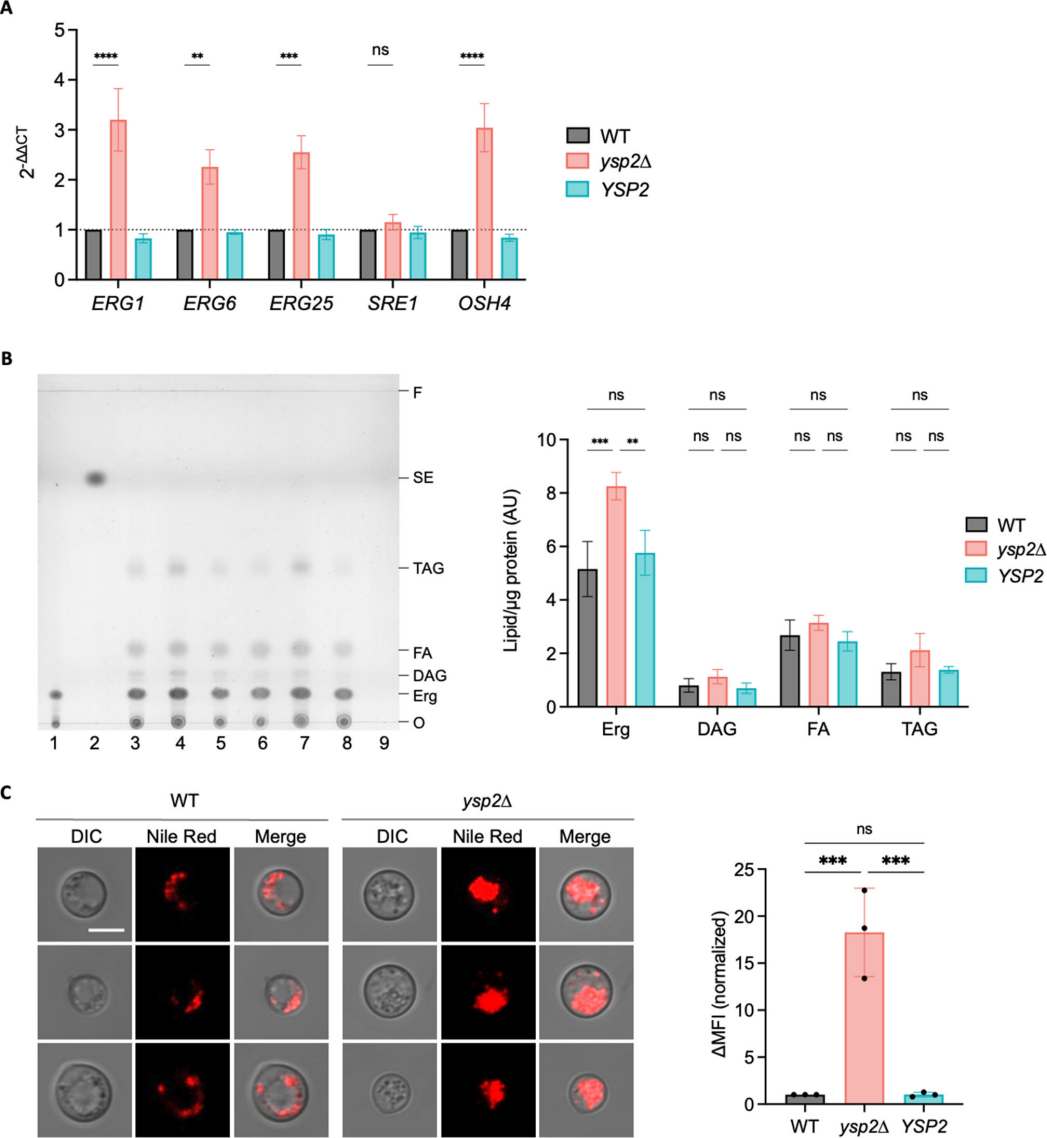
Sterol content, synthesis, and distribution. (**A**) Expression of ergosterol-related genes, measured by RT-qPCR and normalized to *ACT1* expression and WT values. The mean ± SEM of three independent experiments is shown. ns, not significant. ****P < 0.0001, ***P < 0.001, and **P < 0.01 by one-way analysis of variance. (**B**) Lipid profile assessed by thin layer chromatography. Left, a representative thin layer chromatograph, showing two independent biological replicate sample sets. Lane 1, 5 µg/mL ergosterol standard; lane 2, 10 µg/mL cholesteryl oleate standard; lanes 3 and 6, WT; lanes 4 and 7, *ysp2*∆; lanes 5 and 8, *YSP2*; 9, vehicle control. O, origin; F, solvent front; Erg, ergosterol; DAG, diacylglycerols; FA, fatty acids; TAG, triacylglycerols; SE, steryl esters. Right, relative lipid abundance. Mean ± SEM of cellular lipids [identified as in reference (41)], measured by densitometry and normalized to total protein (Fig. S6A) are shown for four independent experiments. ***P < 0.001, and **P < 0.01 by one-way analysis of variance. (**C**) Nile Red staining. Left, representative fluorescence images of WT and mutant strains. All images are to the same scale; bar, 5 µm. Right, ∆MFI (change in median fluorescent intensity) from flow cytometry profiles of the indicated strains. Mean ± SD, normalized to WT, is shown for three independent experiments. **P < 0.01 by one-way analysis of variance.

Our staining experiments suggested that cells lacking Ysp2 have abnormally high PM ergosterol. This is an expected result of reduced retrograde transport from the PM to the ER but may also reflect increased sterol synthesis secondary to decreased ergosterol in the ER. To test the latter idea, we examined whether expression of sterol-related genes, chosen to represent various branches of sterol synthesis and transport, was altered in *ysp2*∆ cells. We detected modest upregulation of genes whose products act in ergosterol synthesis [*ERG1, ERG6,* and *ERG25* (6, 34)] and in transport of ergosterol away from its site of synthesis in the ER [*OSH4* (13, 29, 35, 36)] (Fig. 6A). The expression of *SRE1*, whose product regulates ergosterol synthesis but is itself regulated post-transcriptionally (37 - 40), was not affected (Fig. 6C).

To test whether the changes in gene expression we observed were manifested in ergosterol synthesis, we surveyed neutral lipids by thin layer chromatography (TLC). We found that while *ysp2*∆ cells had slightly higher amounts of all lipid species [when normalized to total protein (Fig. S6A)], their ergosterol content significantly exceeded that of the WT and complemented strains (Fig. 6B). Together, our results suggest that cells lacking Ysp2 undergo both altered ergosterol distribution and increased synthesis of this lipid. The latter is further supported by our observation of more abundant lipid droplets in *ysp2*∆ than in WT and complemented strains (Fig. 6C; Fig. S6B and C (42); see Discussion).

### Probing Ysp2 with antifungal compounds

**Fig 7:**
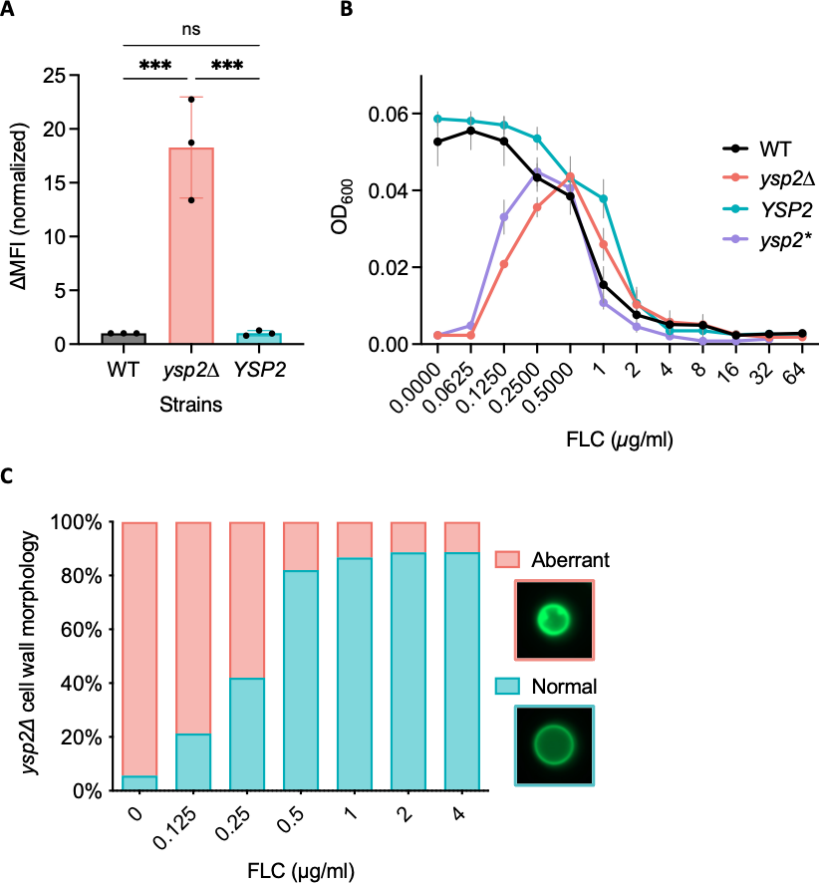
Antifungal drug interactions with *ysp2*∆ cells. (**A**) Amphotericin B-Cy5 binding measured by flow cytometry. Mean ± SD of change in median fluorescent intensity (∆MFI), normalized to WT, is shown for three independent experiments. ns, not significant; ***P < 0.001 by one-way analysis of variance. (**B**) Mutant response to fluconazole (FLC). Cells were grown in 37R5 with FLC as indicated and OD_600_ measured at 48 h. Mean ± SEM of three independent experiments is shown. ysp2*, inactivated mutant. (**C**) Cells were grown in 37R5, and the fraction with aberrant cell walls was quantified at 24 h. Seventy cells were scored per condition.

We previously observed that *ysp2*∆ cells are more sensitive than WT to the antifungal drug AmB (Fig. 3C), which acts by binding and extracting ergosterol (10, 11). We hypothesized that this sensitivity was mediated by their increased cell surface ergosterol, consistent with studies in several other eukaryotic systems (43 - 46). Supporting this idea, these cells bound significantly more Cy5-conjugated AmB than control strains (Fig. 7A; Fig. S7A).

We further hypothesized that the excess PM ergosterol of the mutant cells led to the dramatic surface invaginations we had observed (Fig. 4). If true, the phenotype might be reversed by reducing sterol synthesis. To test this idea, we treated *ysp2*∆ cells with fluconazole, an antifungal that targets lanosterol demethylase (Erg11) and thereby inhibits ergosterol biosynthesis (9). Indeed, low levels of fluconazole showed a striking and dose-dependent rescue of *ysp2*∆ growth, such that it matched WT cell density at 0.5 mg/mL (Fig. 7B). This was accompanied by reversal of the cell wall invagination phenotype (Fig. 7C) and plasma membrane irregularities (Fig. S7B). Notably, *ysp2*∆ cells were not more resistant to fluconazole than WT, since at higher drug concentrations the growth of all strains was similarly reduced (Fig. 7B). This contrasts with a previous study that showed *ysp2*∆ had slightly lower MIC for fluconazole (23), a difference we attribute to the distinct growth conditions used. We consistently observe more dramatic phenotypes (PM and cell wall morphologies) when we grow *ysp2*∆ cells in tissue culture conditions versus rich medium, suggesting that fungal pathogens have a greater need for effective sterol organization in the host environment.

## DISCUSSION

Ergosterol, the major sterol of fungal membranes, is a key player in cell physiology and signal transduction and is also an important target of anticryptococcal drugs. Despite its importance, sterol organization in the context of pathogenesis has remained unexplored. In this study, we determined how Ysp2, a cryptococcal sterol transporter of the LAM family, impacts the ability of cryptococci to maintain key cellular structures, survive in the mammalian host, and cause disease. We found that *ysp2*∆ cells grown in host-like conditions present with striking invaginations of both the PM and the cell wall, as well as increased levels of ergosterol at the PM (Fig. 4).

**Fig 8:**
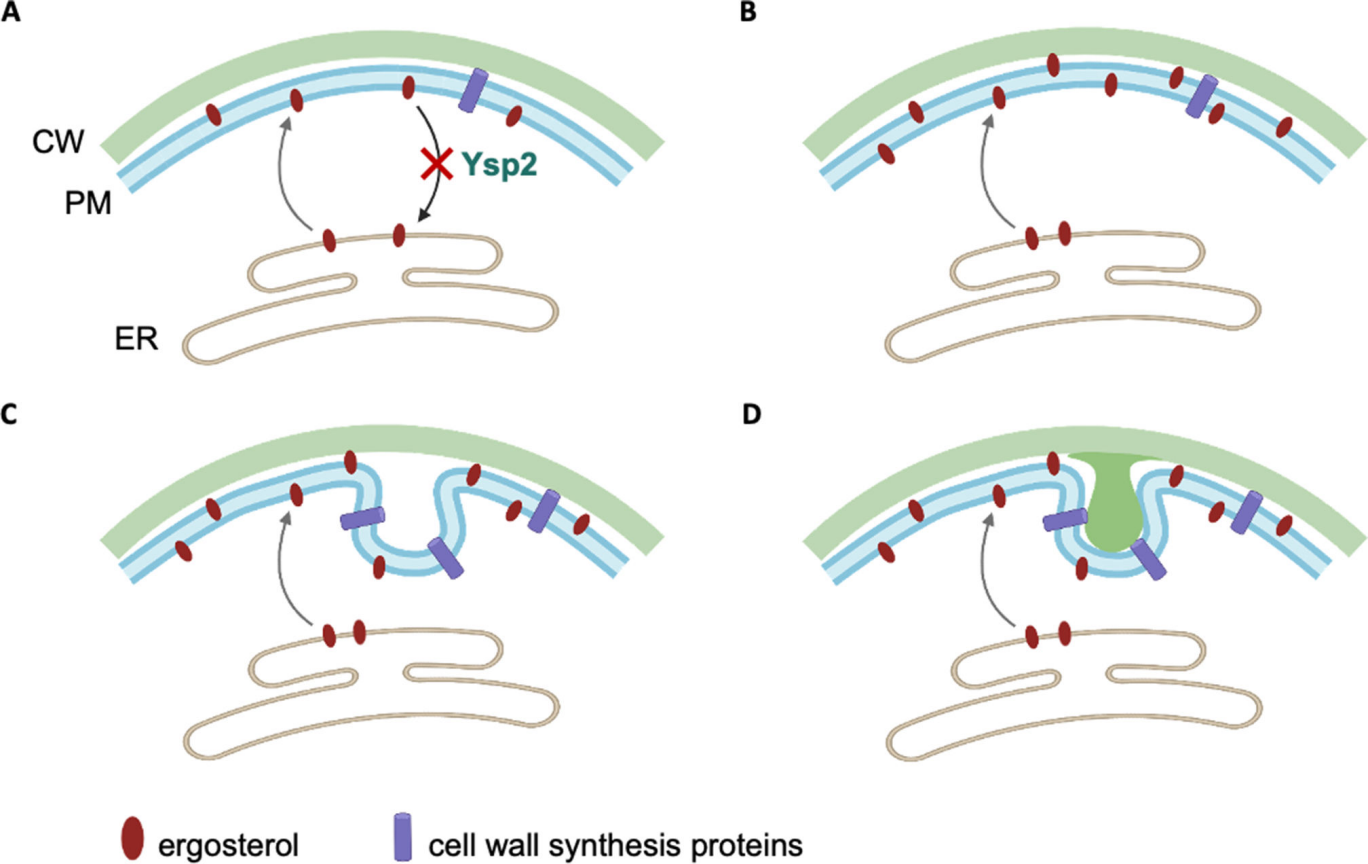
Model of Ysp2 function and how its absence influences plasma membrane and cell wall morphology. CW, cell wall.

At the ER, WT *C. neoformans* cells synthesize ergosterol, which is then transported to the PM by a yet-to-be identified anterograde sterol transporter. We propose that Ysp2 functions as a retrograde transporter, removing excess ergosterol from the PM to balance the system. In the host, there is likely an increased need for efficient ergosterol redistribution to rapidly respond to environmental stress. Based on our observations, we hypothesize that when Ysp2 is absent in this situation, retrograde transport of sterol is abrogated while ergosterol synthesis increases and anterograde transport continues (Fig. 8A), leading to the accumulation of ergosterol at the PM (Fig. 8B). The excess ergosterol causes the PM to invaginate (Fig. 8C), likely by increasing membrane fluidity (47). This in turn causes mislocalization of PM-resident proteins, including proteins involved in cell wall synthesis, and results in enriched synthesis of new cell wall material (darker green in Fig. 8D) within the invaginated areas.

Our evidence for protein mislocalization comes in part from studies of Pma1, a PM ATPase that is associated with lipid rafts (33, 48, 49). Lipid rafts require tight associations of sterols with sphingolipids (32); we speculate that ergosterol accumulation at the PM perturbs these structures, resulting in aberrant protein localization (50 - 52). In support of this idea, Pma1 in the *ysp2*∆ mutant appeared as puncta at the cell surface and occasionally in vacuoles (Fig. 5A; Fig. S5B), in contrast to its smooth PM distribution in WT cells (53). We expect that changes in membrane organization also affect the localization of other PM-resident proteins, such as phospholipase B1 (Plb1), a virulence factor (41), and multiple cell wall synthesis proteins [e.g., Chs3, Cda1, Cda2, and Fks1 (54 - 57)]. The latter group is particularly critical during cell growth in host-like conditions, when fungi actively remodel and thicken their cell walls (Fig. 4C) in response to changes in their environment (58 - 60). We hypothesize that mislocalization of such proteins leads to the aberrant wall synthesis suggested in our model (Fig. 8).

In addition to cell wall alterations, we noted altered capsule architecture in *ysp2*∆ cells, which exhibited both reduced capsule thickness and increased capsule permeability. These phenotypic alterations may be due to changes in two compartments that are impacted by sterol organization: the cell wall and the ER. The protein mislocalization that occurs in *ysp2*∆ cells, mentioned above, likely changes cell wall composition and structure, an idea supported by the changes in chitin revealed by our CFW staining (Fig. 4D). The combination of PM and wall changes may further lead to the mislocalization of capsule attachment and remodeling proteins (e.g., Ags1, Pbx1, or Lhc1) and/or altered ability to attach capsule fibers (58, 59, 61 - 63).

Other potential explanations for the capsule alterations observed in *ysp2∆* cells relate to ER function. Because this organelle is the site of ergosterol synthesis and esterification, loss of retrograde ergosterol transfer will indirectly change ER ergosterol levels, potentially influencing secretory processes and thus the export of capsular polysaccharides. This idea is supported by a previous study, which showed reduced secretion of capsule polysaccharides in a mutant defective in ER homeostasis (64). Changes in ER lipid composition could also alter the localization or activity of ER-resident nucleotide sugar transporters, such as Uut1 and Uxt2, which are required for capsule synthesis (65, 66). Notably, proteins involved in ergosterol synthesis are not strongly implicated in capsule formation: the ergosterol synthesis mutant *erg6*∆ has normal capsule, although the synthesis regulator mutant *sre1*∆ does show slight capsule defects (34, 37, 38, 40). Overall, our results suggest a connection between ergosterol organization and capsule elaboration, which warrants further exploration.

High levels of ergosterol at the PM could potentially be explained by increased synthesis of this compound or aberrant distribution of normal amounts. Based on the putative role of Ysp2 as a sterol transporter, we initially assumed that the latter explained the phenotypes we observed. Consistent with this idea, our microscopy and flow analyses showed increased cell surface ergosterol. Intriguingly, our TLC and qPCR data suggested a more complex story, since *ysp2*∆ cells grown in a host-like environment showed both increased ergosterol and the upregulation of multiple genes involved in its synthesis (Fig. 6A and B). We speculate that this stimulation is triggered by the relative lack of ergosterol *within* the cell, which occurs because mutant cells cannot retrieve it from the PM. Precedent for this idea comes from studies of the *Schizosaccharomyces pombe* Scp1-Sre1 complex, which is activated by depletion of intracellular ergosterol (67). We suggest a similar mechanism for the cryptococcal sterol regulator, Sre1 (37). The resulting increased ergosterol synthesis, although it represents an effort by the cell to maintain homeostasis, may in fact contribute to the skewed sterol distribution caused by reduced retrograde transport, highlighting the complex regulatory pathways that govern sterol organization.

Disrupted lipid homeostasis caused by both increased synthesis and reduced turnover from the PM can be further manifested in other phenotypes. The dramatic increase in lipid droplets that we observe in the mutant is one example (Fig. 6C). In *S. cerevisiae*, excess sterols are not degraded; instead, they are either esterified and stored in lipid droplets, or secreted into the environment as sterol acetates (6). If similar events occur in *C. neoformans*, the increase in lipid droplets likely reflects the esterification and storage of sterols made in response to upregulation of ergosterol synthesis. Beyond esterification, excess sterols may undergo other modifications, such as glycosylation to form sterylglucosides. This is of particular interest in *C. neoformans*, as accumulation of these species leads to reduced fungal virulence (68 - 70).

Multiple factors likely contribute to the reduced virulence of *ysp2*∆. One is the poor growth of this mutant in host-like conditions (Fig. 2D; Fig. S2). Its inability to survive within host phagocytes may also reduce virulence by impeding cryptococcal dissemination in the host (25, 71). The changes that occur in both the capsule and cell wall may further contribute to the reduced virulence, as these are well-characterized virulence factors with critical roles in protecting fungal cells from external stresses (27). A last possibility is that accumulation of ergosterol at the mutant PM affects the host immune response, as work in several fungal pathogens has shown that the level of cell surface ergosterol correlates with the ability of fungal pathogens to trigger host pyroptosis (72, 73).

We have focused on the *C. neoformans* homolog of the *S. cerevisiae* LAM family protein Ysp2. Curiously, *C. neoformans* encodes only one LAM homolog, although *S. cerevisiae* has six such proteins with overlapping activities (Fig. 1A) (19, 74). It may be that cryptococcal Ysp2 serves additional functions compared to the *S. cerevisiae* protein. This idea is supported by the distinct localization of Ysp2 in the two organisms. In the model yeast, Ysp2 is an ER-resident protein that localizes to ER-PM contact sites at the cell periphery (19, 21), while other LAM family members act at ER-PM, ER-mitochondria, or ER-vacuole contact sites (19, 20). In contrast, we observed both intracellular and peripheral localization of *C. neoformans* Ysp2. This suggests that it acts at additional membrane interfaces, such as those of mitochondria or vacuoles, as well as at surface contact sites. Another possible explanation for the single LAM family protein in *C. neoformans* is that additional proteins involved in *C. neoformans* sterol transport, which lack homology to this family yet perform similar functions, remain to be discovered.

We have identified a retrograde sterol transporter in *C. neoformans*, Ysp2, that is critical for virulence by influencing key cellular functions including PM integrity, cell wall formation, capsule elaboration, and lipid homeostasis. We provide a model that explains the phenotypes observed in *ysp2*∆ and predicts how cells respond to excess ergosterol accumulation. Our findings provide insights into the role of sterol transport in cryptococcal biology, particularly in the context of the host. Beyond the future directions mentioned above, these discoveries suggest multiple important topics that remain to be explored, including the complete set of proteins responsible for sterol transport in *C. neoformans*, how sterol accumulation changes the biophysical properties of the cryptococcal PM, and ergosterol regulation and homeostasis in the environment of the infected host.

## MATERIALS AND METHODS

### Cell growth and strain construction

*C. neoformans* strains were grown overnight in yeast extract-peptone-dextrose (YPD) medium [1% (wt/vol) Bacto yeast extract, 2% (wt/vol) dextrose, 2% (wt/vol) Bacto peptone in double-distilled water (ddH_2_O)] at 30°C with shaking at 230 rpm, collected by centrifugation, washed twice with sterile PBS, diluted to 10^6^ cells/mL in RPMI, and incubated at 37°C in 5% CO_2_ for 24 h in 6-well plates or T-75 tissue culture flasks. For growth curves, cells were grown overnight in YPD, washed, and adjusted to 1 × 10^5^ cells/mL for growth in YPD (30°C, 37°C, or 37°C + 5% CO_2_), RPMI at 37°C + 5% CO_2_ (37R5), or DMEM at 37°C + 5% CO_2_ (37D5).

Strain construction and tagging strategies are detailed in Text S1.

### Virulence studies

All animal protocols were approved by the Washington University Institutional Animal Care and Use Committee (Protocol #20–0108), and care was taken to minimize animal handling and discomfort.

For survival studies, groups of ten 8-week-old female C57BL/6 mice (The Jackson Laboratory, Bar Harbor, ME, USA) were anesthetized by injection of 1.20 mg ketamine and 0.24 mg xylazine in 110  µL sterile PBS and intranasally infected with 1.25  ×  10^4^ cryptococcal cells. The mice were monitored and humanely sacrificed when their weight decreased to below 80% of their initial weight or if they showed signs of disease. To assess organ burden at the time of sacrifice, the lungs and brains were harvested, homogenized, diluted, and plated on YPD agar. The resulting CFUs were enumerated, and survival differences were assessed by Kaplan-Meier analysis.

### Fungal intracellular survival

We obtained bone marrow-derived macrophages (BMDM) as detailed in Text S1. Fungal cells from overnight YPD cultures were washed in PBS, opsonized in 20% human serum in PBS (10^7^ cells/mL, 30 minutes, 37°C), washed in PBS, and resuspended in RPMI. Opsonized fungi were added to BMDM cells at an MOI of 0.1 and incubated for 1.5 h to permit engulfment. Wells were then washed and refilled with prewarmed BMDM medium, and plates were incubated for 0, 24, or 48 h at 37°C, 5% CO_2_; washed twice with sterile PBS; refilled with sterile ddH_2_O; incubated at room temperature for 30 minutes to lyse BMDM; and plated on YPD agar to quantify CFU.

### Microscopy and flow cytometry

For imaging, fungal strains were grown as above but resuspended at 10^7^ cells/mL for staining as detailed in Text S1 and then imaged using a ZEISS Axio Imager M2 fluorescence microscope or a ZEISS LSM880 confocal laser scanning microscope. Electron microscopy was performed as detailed in Text S1. For flow cytometry, cells were resuspended in 1 mL of PBS with 10 mM NaN_3_. Data were acquired on a BD LSRFortessa Cell Analyzer and analyzed using FlowJo software.

### Phenotyping

Cells grown as above were adjusted to 10^7^ cells/mL in PBS and serially diluted to final cell concentrations of 10^6^, 10^5^, 10^4^, and 10^3^ cells/mL. Four microliters of each dilution were spotted onto YPD and stress plates and grown at 30°C and 37°C. To impose membrane stress, YPD agar was supplemented with 0.01% SDS, 1.2 M NaCl, 1 µg/mL amphotericin B, and 8 µg/mL fluconazole. YPD agar was supplemented with 0.2% calcofluor white (wt/vol) and 0.05% Congo Red for cell wall stress, or 0.125 µg/mL tunicamycin for ER stress.

### Sterol analysis

The protocol for lipid extraction was based on (75). Details of lipid extraction and TLC analysis are provided in Text S1.

### qPCR

Total RNA was extracted using TRI-Reagent (Applied Biosystem, Waltham, MA, USA), and cDNAs were synthesized using the SuperScript III First-Strand Synthesis System SuperMix Kit (Invitrogen, Carlsbad, CA, USA) for quantitative PCR analysis using the SYBR Green PCR Master Mix Kit (Applied Biosystems) as recommended by the supplier and a CFX96 Touch Real-Time PCR Detection System (Bio-Rad, Hercules, CA, USA). Relative gene expression was calculated using the CT comparative method (2^−ΔΔCT^), with *ACT1* expression as a normalization control.

## SUPPLEMENTAL MATERIAL

Text S1 mbio.01353-23-s0001.pdf Supplemental methods.

Fig. S1 mbio.01353-23-s0002.pdf Virulence and viability of *ysp2*∆ cells.

Fig. S2 mbio.01353-23-s0003.pdf Growth of *ysp2*∆ cells.

Fig. S3 mbio.01353-23-s0004.pdf Capsule permeability and AmB sensitivity.

Fig. S4 mbio.01353-23-s0005.pdf Mitochondrial functions of *ysp2*∆.

Fig. S5 mbio.01353-23-s0006.pdf Pma1-mNG expression and localization.

Fig. S6 mbio.01353-23-s0007.pdf Protein and lipid droplets in *ysp2*∆.

Fig. S7 mbio.01353-23-s0008.pdf Aberrant surface lipids in *ysp2*∆.

ASM does not own the copyrights to Supplemental Material that may be linked to, or accessed through, an article. The authors have granted ASM a non-exclusive, world-wide license to publish the Supplemental Material files. Please contact the corresponding author directly for reuse.
