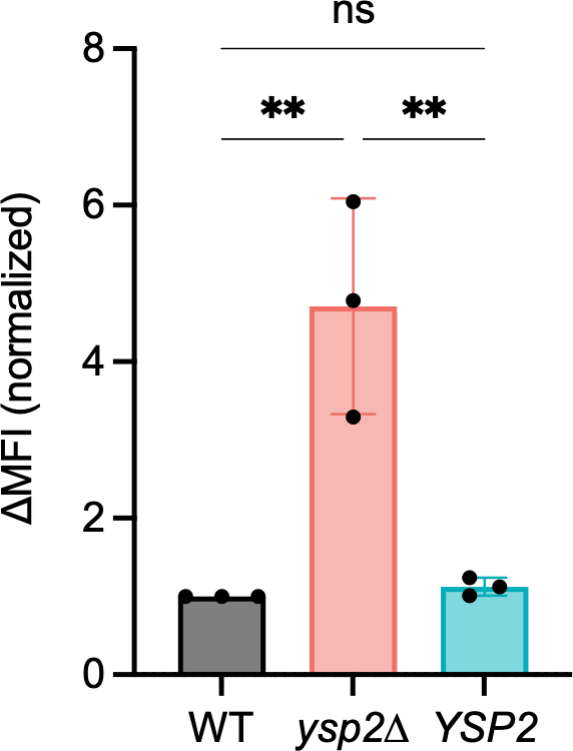# Correction for Choy et al., “Ergosterol distribution controls surface structure formation and fungal pathogenicity”

**DOI:** 10.1128/mbio.03550-24

**Published:** 2024-12-17

**Authors:** Hau Lam Choy, Elizabeth A. Gaylord, Tamara L. Doering

## AUTHOR CORRECTION

Volume 14, no. 4, e01353-23, 2023, https://doi.org/10.1128/mbio.01353-23. Page 6, paragraph starting “Our staining experiments,” last sentence: “Fig. 6C” should read “Fig. 6A.”

Page 9, Fig. 6C: The right panel should appear as shown in this correction. Regretfully, we inadvertently included a plot from Fig. 7A when composing the figure. We apologize for this mistake, which did not change any data interpretation or conclusions of our work.

**Fig 6:**