# Supplementary material for: Ergosterol distribution controls surface structure formation and fungal pathogenicity
Source: mBio. 2023 Jul 6;14(4):e01353-23. doi: 10.1128/mbio.01353-23 (PMC10470819; doi:10.1128/mbio.01353-23)
Supplement: Text S1 — Supplemental methods. [file mbio.01353-23-s0001.pdf]

SUPPLEMENTARY TEXT S1

Strain construction

All *C. neoformans* strains used were in the serotype A KN99α background. For strain construction, we used a split-marker strategy (1) with biolistic transformation, selected candidates by patterns of drug resistance, and confirmed all candidate transformants by PCR and whole genome sequencing. We first generated two *ysp2*Δ deletion strains by replacing *YSP2* with either a G418 (2) or NAT resistance marker (3). To complement these mutants at the native site, we replaced the G418 or NAT coding sequence with the *YSP2* coding sequence, preceded by a NAT or G418 resistance marker, respectively. To endogenously tag *YSP2*, we used a tagging module consisting of codon-optimized mNeonGreen, a tandem Calmodulin Binding Peptide-2X FLAG Tag, and a NAT resistance marker.

To endogenously tag Pma1, we used a CRISPR-Cas9 strategy to tag the C-terminus of Pma1 (*CNAG_06400*) with an mNeonGreen tagging module as described above in tandem with a G418 marker (4).

BMDM Preparation

For bone marrow-derived macrophages (BMDMs), we isolated bone marrow from C57BL/6 mouse femurs and expanded the cellular population by growth in BMDM medium (RPMI supplemented with 10% heat-inactivated FBS and 20% L cell supernatant) for seven days. Macrophages were isolated using anti-F4/80 conjugated biotin and anti-biotin magnetic microbeads.

Microscopy

For imaging, fungal strains were grown as in the main text and resuspended at 10^7 cells/ml for staining with Lucifer Yellow (200 µg/ml, 30 min, RT), calcofluor white, (100 µg/ml, 15 min, RT), filipin (5 µg/ml 10 min, RT), amphotericin B-Cy5 (10 µM, 45 min, 37˚C), or Nile Red (0.0005%, 15 min, RT) (5–7). For capsule imaging, cells were resuspended in 600 µl PBS and mixed with 300 µl India Ink or stained (30 min, RT) with 50 µg/ml of anticapsular monoclonal antibody 302 conjugated to Alexa Fluor 488 (Molecular Probes).

For electron microscopy, cells were grown as described above and fixed in 2.5% glutaraldehyde (Ted Pella Inc., Redding, CA) in 0.1 M sodium cacodylate buffer for 1 h at room temperature and then overnight at 4°C. Samples were washed in sodium cacodylate buffer and postfixed in 1% osmium tetroxide (Ted Pella Inc.) for 1 h at room temperature before dehydration by successive 30 min incubations in water–ethanol mixtures with increasing ethanol concentrations. The ethanol-substituted samples were then substituted in propylene oxide (twice for 30 min each) and infiltrated and embedded in Eponate 12 resin (Ted Pella, Redding, CA). Blocks were polymerized overnight at 65°C. Ultrathin sections of 95 nm were cut with a Leica Ultracut UCT ultramicrotome (Leica Microsystems, Bannockburn, IL), stained with uranyl acetate and lead citrate, and viewed on a JEOL 1200 EX transmission electron microscope (JEOL USA Inc., Peabody, MA) equipped with an AMT 8-megapixel digital camera and AMT Image Capture Engine V602 software (Advanced Microscopy Techniques, Woburn, MA).

Sterol Analysis

Lipid extraction was performed as previously described with slight modifications (8). Briefly, 5 x 10^8 cells were washed twice in sterile water and resuspended in 1 ml chloroform: 0.8 ml dH_2O: 2 ml methanol. Samples were vortexed, sonicated (Fisher Scientific Sonic Dismembrator Model 300; 40% power for 30 s) three times at intervals of 30 s, and incubated at 60˚C for 15 min. This process was repeated, cell debris was removed by centrifugation, and the supernatant fraction was evaporated and stored at -20˚C.

For TLC analysis, lipid extracts were resuspended in 100 µl of chloroform-methanol (2:1), spotted (20 µl) on silica plates along with standards (5 µg ergosterol and 10 µg of cholesteryl oleate), and the plates were developed in petroleum ether-diethyl ether-acetic acid (80:20:1 [vol/vol/vol]) (9). Total lipids were visualized by treatment with sterol spray (50% ethanol in water, 3.2% H_2SO_4, and 0.5% $MnCl_2$) followed by 30 minutes of charring at 120˚C (10). Densitometry was performed using ImageJ software.

References

1. Fu J, Hettler E, Wickes BL. 2006. Split marker transformation increases homologous integration frequency in *Cryptococcus neoformans*. Fungal Genetics and Biology 43:200–212.

2. Hua J, Meyer JD, Lodge JK. 2000. Development of Positive Selectable Markers for the Fungal Pathogen *Cryptococcus neoformans*. Clin Diagn Lab Immunol 7:125–128.

3. McDade HC, Cox GM. 2001. A new dominant selectable marker for use in *Cryptococcus neoformans*. Medical Mycology 39:151–154.

4. Huang MY, Joshi MB, Boucher MJ, Lee S, Loza LC, Gaylord EA, Doering TL, Madhani HD. 2022. Short homology-directed repair using optimized Cas9 in the pathogen *Cryptococcus neoformans* enables rapid gene deletion and tagging. Genetics 220:iyab180.

5. Thoms S, Debelyy MO, Connerth M, Daum G, Erdmann R. 2011. The Putative *Saccharomyces cerevisiae* Hydrolase Ldh1p Is Localized to Lipid Droplets. Eukaryotic Cell 10:770–775.

6. Nolan SJ, Fu MS, Coppens I, Casadevall A. 2017. Lipids Affect the *Cryptococcus neoformans*-Macrophage Interaction and Promote Nonlytic Exocytosis. Infect Immun 85:e00564-17.

7. Kohlwein SD, Veenhuis M, van der Klei IJ. 2013. Lipid Droplets and Peroxisomes: Key Players in Cellular Lipid Homeostasis or A Matter of Fat—Store ’em Up or Burn ’em Down. Genetics 193:1–50.

8. Singh A, MacKenzie A, Girnun G, Del Poeta M. 2017. Analysis of sphingolipids, sterols, and phospholipids in human pathogenic *Cryptococcus* strains. J Lipid Res 58:2017–2036.

9. Siafakas AR, Wright LC, Sorrell TC, Djordjevic JT. 2006. Lipid Rafts in *Cryptococcus neoformans* Concentrate the Virulence Determinants Phospholipase B1 and Cu/Zn Superoxide Dismutase. Eukaryot Cell 5:488–498.

10. Knittelfelder OL, Kohlwein SD. 2017. Thin-Layer Chromatography to Separate Phospholipids and Neutral Lipids from Yeast. Cold Spring Harb Protoc 2017:pdb.prot085456.
