# Supplementary material for: Ergosterol distribution controls surface structure formation and fungal pathogenicity
Source: mBio. 2023 Jul 6;14(4):e01353-23. doi: 10.1128/mbio.01353-23 (PMC10470819; doi:10.1128/mbio.01353-23)
Supplement: Fig. S1 — Virulence and viability of ysp2∆ cells. [file mbio.01353-23-s0002.pdf]

A

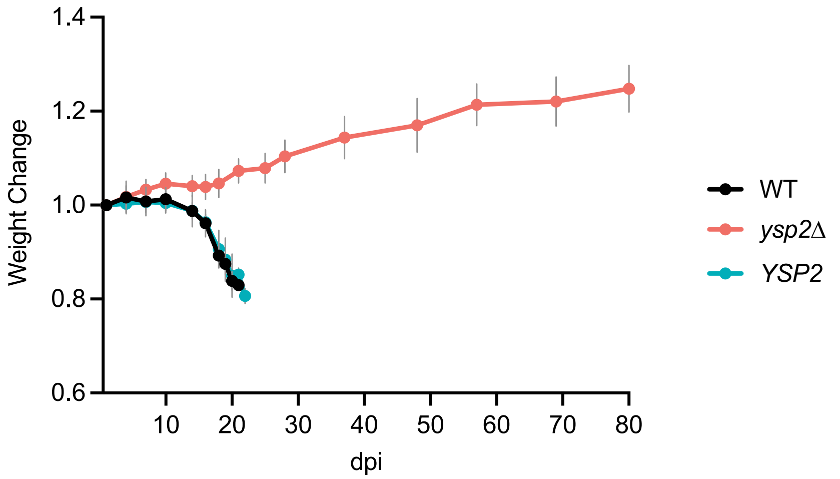

B

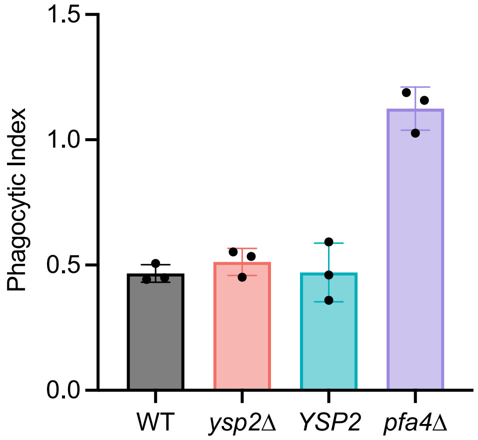

Fig S1. Virulence and viability of *ysp2*Δ cells. (A) Weight of mice infected with the indicated strains over time, normalized to initial weight. Mean ± SD are displayed for 10 mice per group. (B) Phagocytic index (internalized fungi/host cells) of the indicated strains after 1 h incubation as described in the Methods. *pfa4*Δ, positive control strain for increased phagocytic index (51). Mean ± SD of three independent experiments is shown.
