# Supplementary material for: Ergosterol distribution controls surface structure formation and fungal pathogenicity
Source: mBio. 2023 Jul 6;14(4):e01353-23. doi: 10.1128/mbio.01353-23 (PMC10470819; doi:10.1128/mbio.01353-23)
Supplement: Fig. S2 — Growth of ysp2∆ cells. [file mbio.01353-23-s0003.pdf]

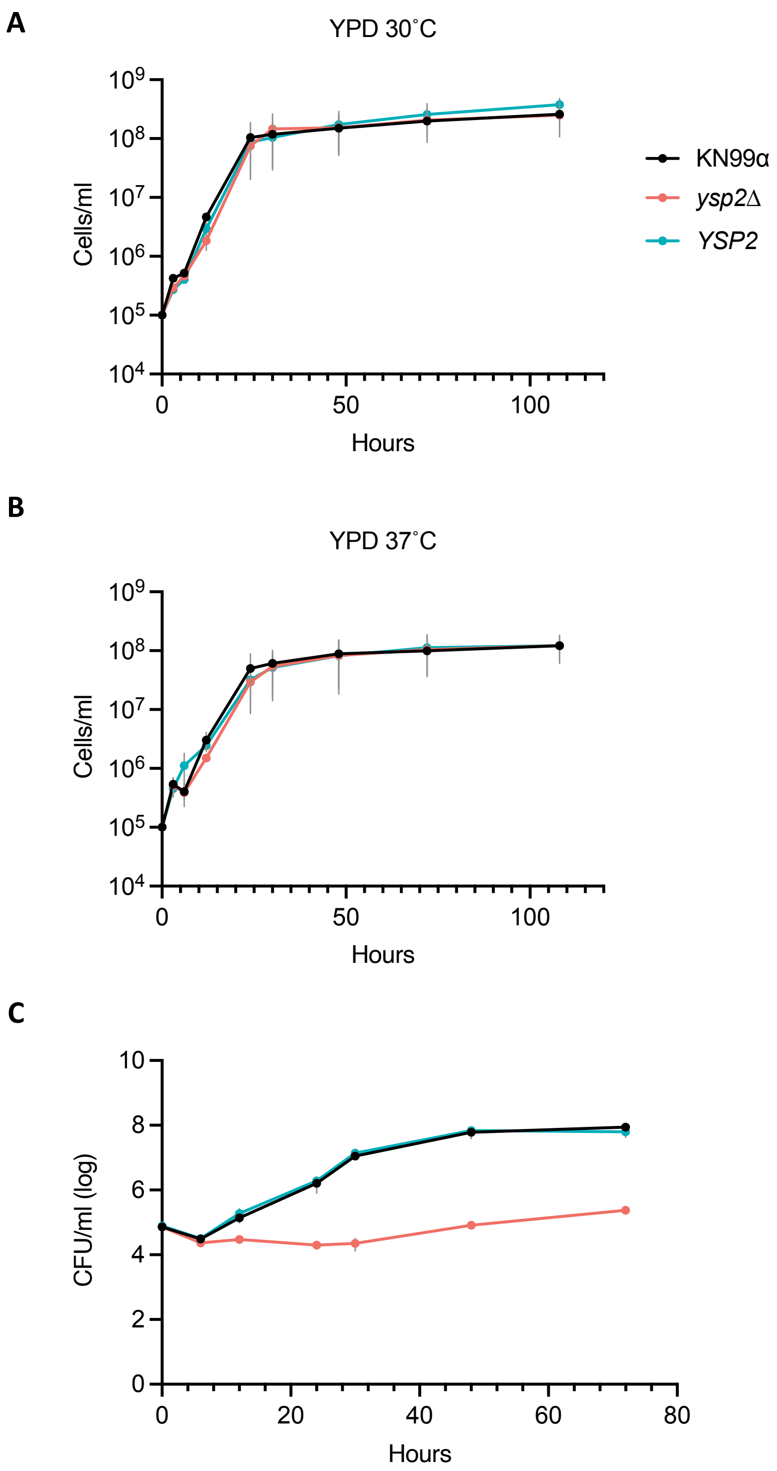

Fig S2. Growth of *ysp2*Δ cells. (A and B) Growth of the indicated strains in the conditions shown (mean ± SEM of three independent experiments). (C) Cells grown in 37R5 were sampled at the times indicated and plated on YPD to assess viability (measured as number of CFUs).
