# Supplementary material for: Ergosterol distribution controls surface structure formation and fungal pathogenicity
Source: mBio. 2023 Jul 6;14(4):e01353-23. doi: 10.1128/mbio.01353-23 (PMC10470819; doi:10.1128/mbio.01353-23)
Supplement: Fig. S3 — Capsule permeability and AmB sensitivity. [file mbio.01353-23-s0004.pdf]

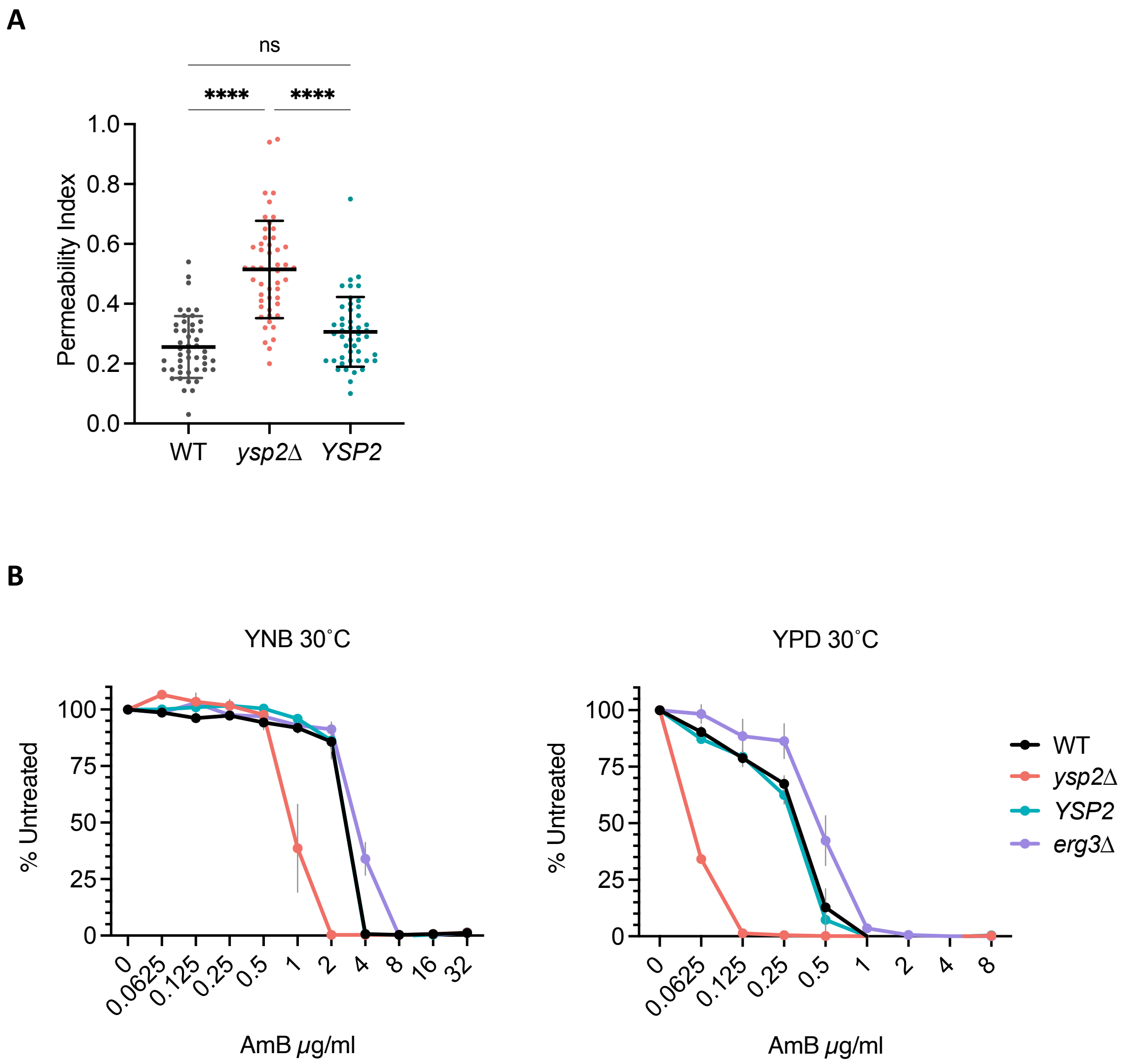

Fig S3. Capsule permeability and AmB sensitivity. (A) Capsule permeability of the indicated strains measured as the fraction of the capsule radius (defined by India ink exclusion) that is penetrated by 2,000 kDa Dextran beads. >40 cells were quantified per sample, plotted with mean ± SD. (B) Cells were grown in the indicated conditions with AmB added as shown. OD600 was measured at 48 h and plotted relative to that of the untreated control. Mean ± SEM of three independent experiments is shown. erg3Δ, a control strain with lower ergosterol levels (73).
