# Supplementary material for: Ergosterol distribution controls surface structure formation and fungal pathogenicity
Source: mBio. 2023 Jul 6;14(4):e01353-23. doi: 10.1128/mbio.01353-23 (PMC10470819; doi:10.1128/mbio.01353-23)
Supplement: Fig. S4 — Mitochondrial functions of ysp2∆. [file mbio.01353-23-s0005.pdf]

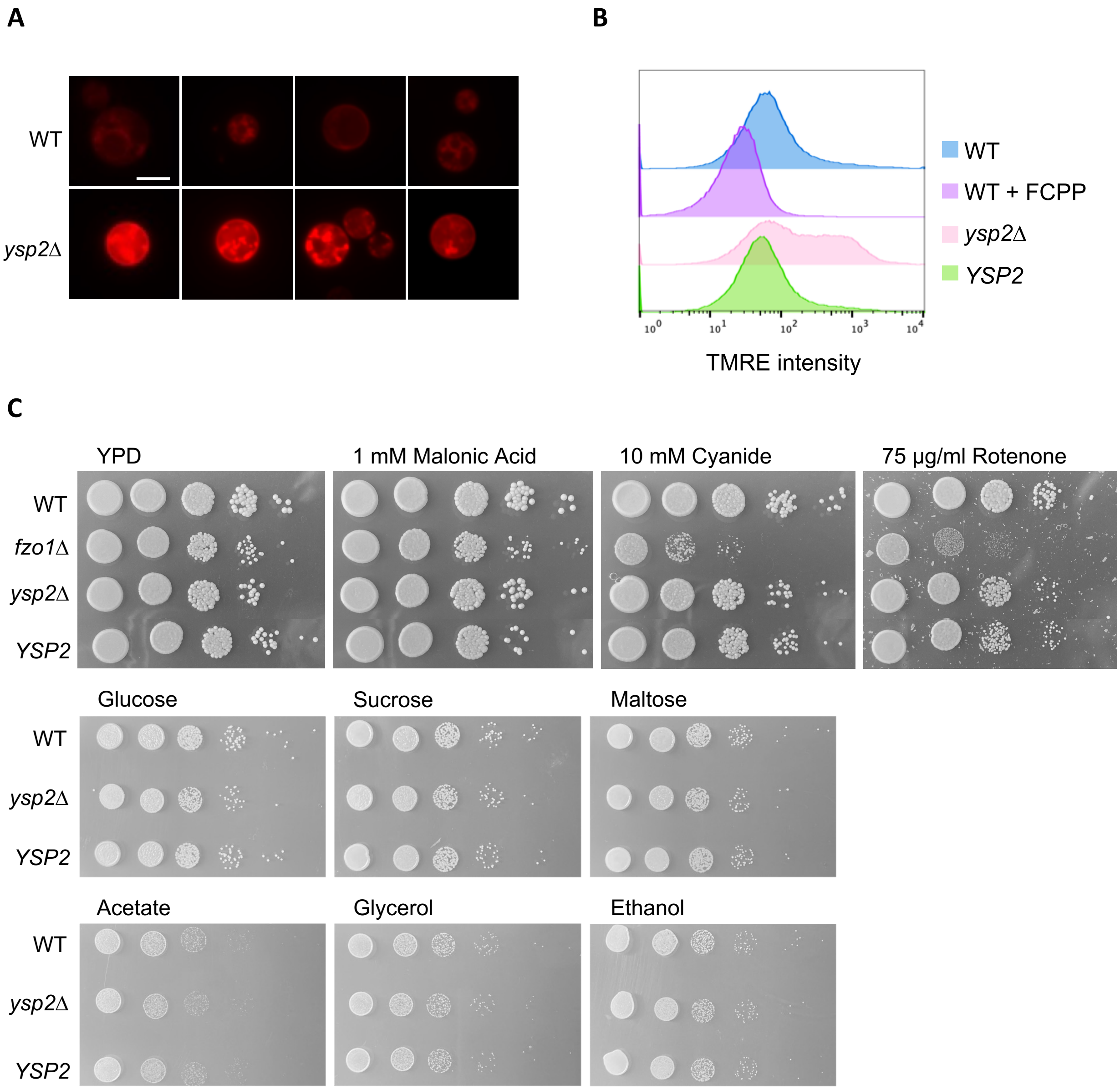

Fig S4. Mitochondrial functions of *ysp2Δ*. (A) Representative images of cells grown in 37D5 + 1 mM H_2O_2 stained with 1 mM MitoTracker CMXRos. All images are captured with the same parameters and shown to the same scale; bar, 5 µm. (B) TMRE fluorescence intensity quantified by flow cytometry of the indicated strains after 24 h growth in YPD. FCPP, carbonyl cyanide 4-(trifluoromethoxy) phenylhydrazone, an electron transport chain uncoupler. (C) Stress phenotypes of the indicated strains on rich medium (YPD). Serial dilutions of the indicated strains were grown in the absence or presence of the indicated additives. *fzo1Δ*, control strain for impaired mitochondrial function (74). Alternative carbon sources were tested at 2% (wt/vol).
