# Supplementary material for: Ergosterol distribution controls surface structure formation and fungal pathogenicity
Source: mBio. 2023 Jul 6;14(4):e01353-23. doi: 10.1128/mbio.01353-23 (PMC10470819; doi:10.1128/mbio.01353-23)
Supplement: Fig. S5 — Pma1-mNG expression and localization. [file mbio.01353-23-s0006.pdf]

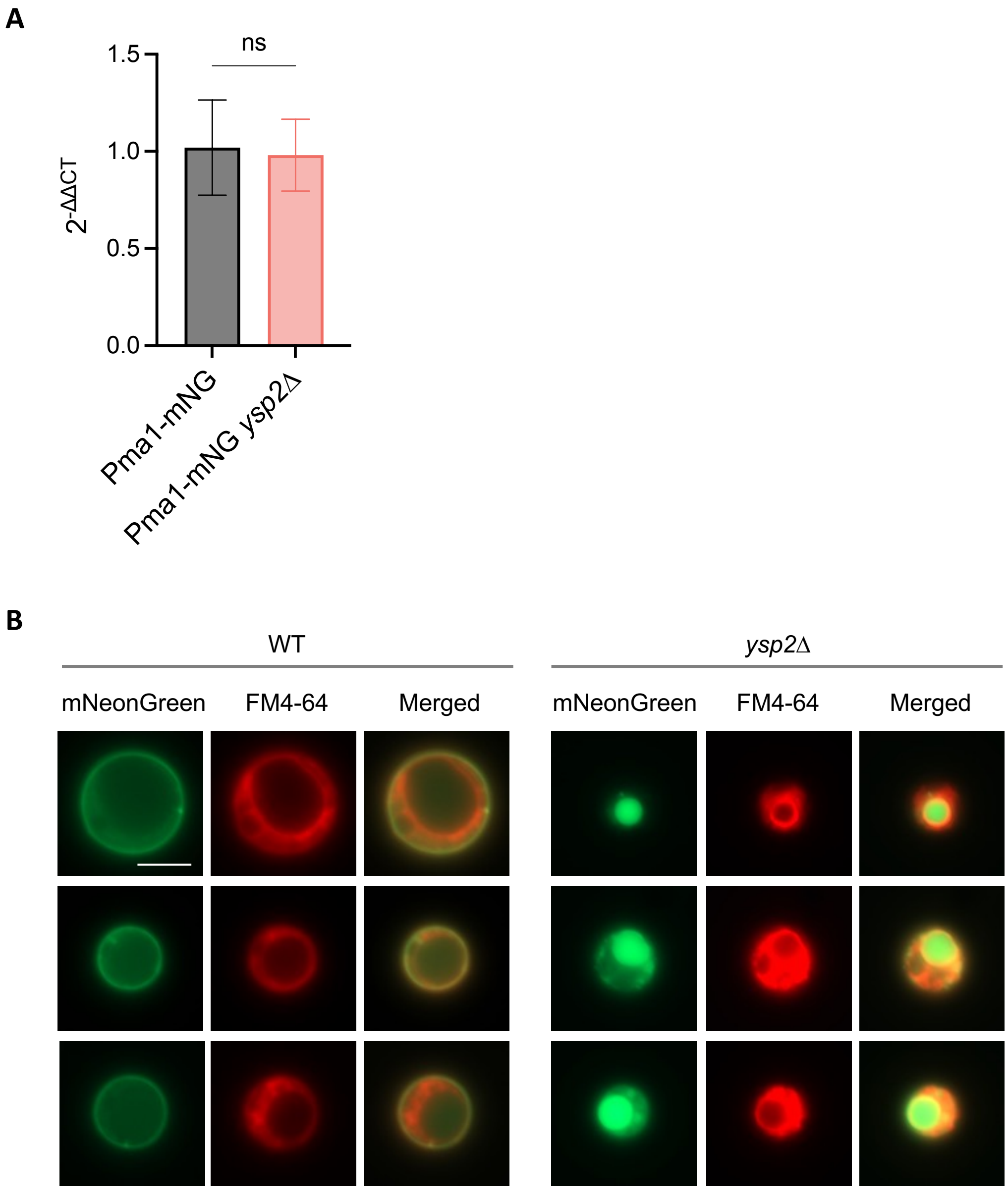


**Fig S5.** Pma1-mNG expression and localization. (A) *PMA1* expression measured by RT-qPCR and normalized to *ACT1* expression and WT values. The mean ± SEM of three biological replicates is shown. (B) Representative fluorescence micrographs of WT and mutant strains with mNeonGreen-tagged Pma1 and the vacuole counterstained with FM4-64. All images are to the same scale; bar, 5 µm.
