# Supplementary material for: Ergosterol distribution controls surface structure formation and fungal pathogenicity
Source: mBio. 2023 Jul 6;14(4):e01353-23. doi: 10.1128/mbio.01353-23 (PMC10470819; doi:10.1128/mbio.01353-23)
Supplement: Fig. S6 — Protein and lipid droplets in ysp2∆. [file mbio.01353-23-s0007.pdf]

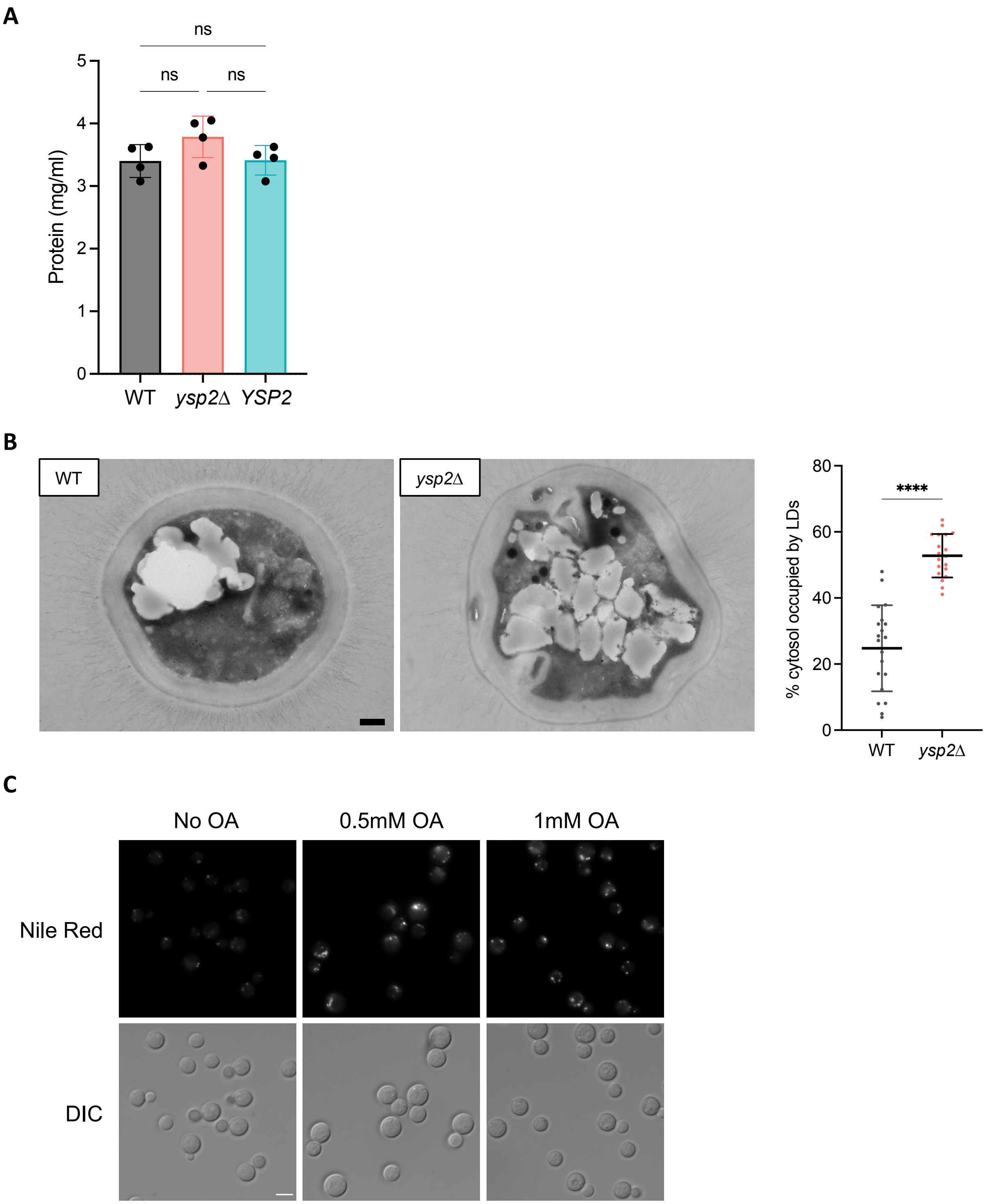

Fig S6. Protein and lipid droplets in *ysp2*Δ. (A) BCA protein quantification for 5 x 10^8 cells of the indicated strains, used to normalize lipid analysis by TLC. The mean ± SD of four biological replicates is shown. (B) Left, transmission electron micrographs of cells grown in 37D5, showing irregular electron-lucent lipid droplets (LD). Both images are to the same scale; bar, 500 nm. Right, percent of the cytosol occupied by LDs in EM sections like those at left. Mean ± SD of at least 20 cells per strain is shown. (C) Representative images of cells grown with the indicated concentrations of oleic acid (OA) and stained with Nile Red to show lipid droplets (75).
