# Supplementary material for: Ergosterol distribution controls surface structure formation and fungal pathogenicity
Source: mBio. 2023 Jul 6;14(4):e01353-23. doi: 10.1128/mbio.01353-23 (PMC10470819; doi:10.1128/mbio.01353-23)
Supplement: Fig. S7 — Aberrant surface lipids in ysp2∆. [file mbio.01353-23-s0008.pdf]

A

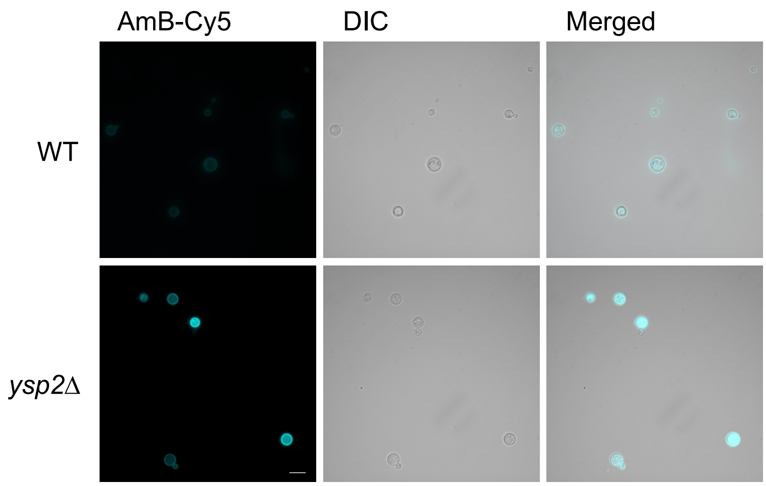

B

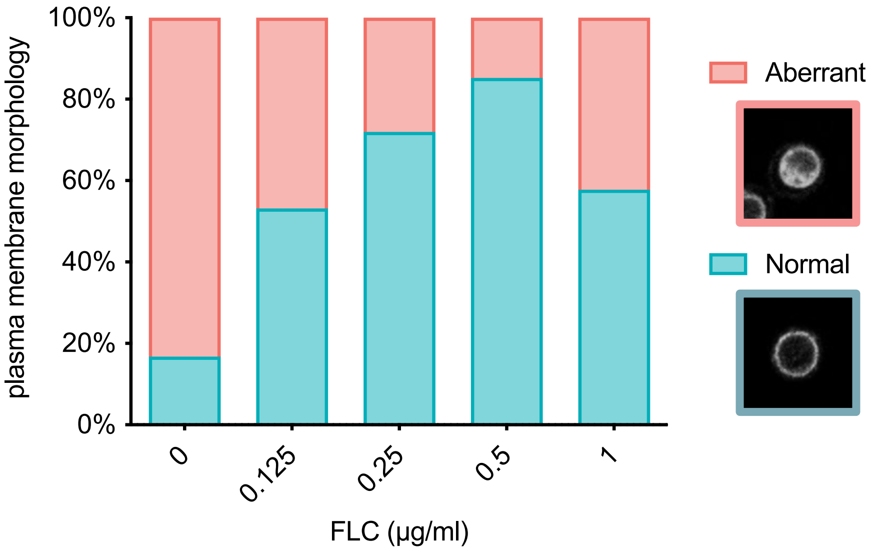

Fig S7. Aberrant surface lipids in *ysp2Δ.* (A) Representative fluorescence images of cells stained with 10 µM AmB-Cy5. All images are to the same scale; bar, 10 µm. (B) Rescue of *ysp2Δ* plasma membrane invagination by fluconazole. Cells were grown in 37R5 and the fraction of aberrant cell membranes quantified at 24 h. 30 cells were scored per condition.
